# Dietary characteristics associated with the risk of non‐alcoholic fatty liver disease and metabolic dysfunction‐associated steatotic liver disease in non‐obese Japanese participants: A cross‐sectional study

**DOI:** 10.1002/jgh3.13082

**Published:** 2024-05-22

**Authors:** Hirokazu Taniguchi, Miho Ueda, Fumika Sano, Yukiko Kobayashi, Takatomo Shima

**Affiliations:** ^1^ Division of Applied Life Sciences, Graduate School of Life and Environmental Sciences Kyoto Prefectural University Kyoto Japan; ^2^ Center for Health Promotion, Japanese Red Cross Kyoto Daiichi Hospital Kyoto Japan

**Keywords:** dietary characteristics, metabolic dysfunction‐associated steatotic liver disease, non‐alcoholic fatty lever disease, non‐obese MASLD, non‐obese NAFLD

## Abstract

**Background and Aim:**

Dietary characteristics associated with non‐alcoholic fatty liver disease (NAFLD) and metabolic dysfunction‐associated steatotic liver disease (MASLD) in non‐obese patients remain to be elucidated. This study examined the association of NAFLD and MASLD with dietary characteristics according to obesity status.

**Methods:**

We performed a cross‐sectional study of 15 135 participants (*n* = 7568 men and 7567 women) aged 35–74 years using data of annual health checks between 2008 and 2020. Obesity was defined as BMI ≥ 25 kg/m^2^. Diagnosis of fatty liver was based on abdominal ultrasonography. Fatty‐liver‐related dietary characteristics were assessed using a self‐administered questionnaire.

**Results:**

For non‐obese participants, NAFLD was found in 31.0% of men and 19.4% of women. Non‐obese MASLD was found in 27.6% of men and 18.1% of women. Multivariable‐adjusted stepwise logistic regression analysis indicated that, in males, both non‐obese NAFLD and non‐obese MASLD were significantly and negatively associated with “often eat sesame/nuts”, and positively associated with “often eat noodles/rice bowl” and “often eat evening meal” (*P* < 0.05). For non‐obese women, both NAFLD and MASLD were significantly and positively associated with “often eat sweet buns/bread with fillings” (*P* < 0.05). Adjusted analyses showed that all dietary characteristics were not significantly associated with the risk of NAFLD/MASLD in obese men and women.

**Conclusion:**

This cross‐sectional study indicates the existence of sex and obesity differences in the association of NAFLD and MASLD with dietary characteristics. Our findings suggest that some dietary characteristics are associated with NAFLD and MASLD prevalence in non‐obese Japanese participants.

## Graphical abstract

Some dietary characteristics are associated with the prevalence of non‐alcoholic fatty liver disease (NAFLD) and metabolic dysfunction‐associated steatotic liver disease (MASLD) in non‐obese participants.The findings suggest the importance of healthy dietary choices to prevent non‐obese steatotic liver disease. Furthermore, the dietary characteristics are not identical between men and women. The results suggest that it is necessary to pay attention to sex differences in dietary characteristics to prevent non‐obese steatotic liver disease.

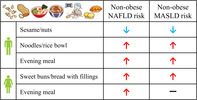

## Introduction

Non‐alcoholic fatty liver disease (NAFLD) is the most prevalent liver disease in the world, and is associated with increased risks of hepatocellular carcinoma and cardiovascular events.^1^, ^2^ NAFLD is generally related to obesity^3^; however, the global prevalence of non‐obese NAFLD has been recently described.^4^, ^5^, ^6^, ^7^ Previous studies have reported that patients with non‐obese NAFLD have higher comorbidities and mortality than those with obese NAFLD.^8^, ^9^ Therefore, it is important to examine the underlying risk factors associated with the morbidity of non‐obese NAFLD.

NAFLD is associated with obesity‐related dietary characteristics such as fast eating,^10^, ^11^ consumption of soft drinks,^12^, ^13^ and consumption of snacks.^14^ In the case of food choice and nutrients, it has been found that the risk of NAFLD is lowered by the intake of high‐fiber foods and unsaturated fatty acids.^15^ A previous study of non‐obese Korean adults reported that the risk of NAFLD was increased in subjects who ate fast.^16^ A study in Japanese non‐obese male adolescents reported that NAFLD was associated with the consumption of soft drinks.^17^ Although these results suggest that dietary characteristics are independent risk factors for non‐obese NAFLD, the dietary characteristics that are key features of non‐obese NAFLD remain to be elucidated. Additionally, sex differences in NAFLD have been documented^18^; however, the relationship between sex and dietary characteristics in non‐obese NAFLD remains to be investigated.

Recently, an international consensus panel proposed novel criteria of fatty liver, instead of NAFLD.^19^, ^20^ For the nomenclature, the name chosen to replace NAFLD was “metabolic dysfunction‐associated steatotic liver disease” (MASLD), which is defined as liver steatosis with at least one of five cardiometabolic risk factors.^19^, ^20^ The risk factors are obesity/abdominal obesity, impaired glucose levels, hypertension, hypertriglyceridemia, and low levels of high‐density lipoprotein (HDL) cholesterol.^19^, ^20^ According to the new criteria, obese patients with NAFLD are classified as obese MASLD, whereas non‐obese patients with NAFLD are classified as non‐MASLD or MASLD. Therefore, we were additionally concerned about the association between dietary characteristics and non‐obese MASLD, which has important implications for the prevention of MASLD.

This study aimed to examine the association between dietary characteristics and NAFLD according to obesity status and sex, thereby identifying NAFLD‐related dietary characteristics in non‐obese participants. Additionally, a secondary analysis was conducted to determine whether the dietary association was comparable among the non‐obese MASLD participants.

## Methods

### Study design and participants

**Figure 1:**
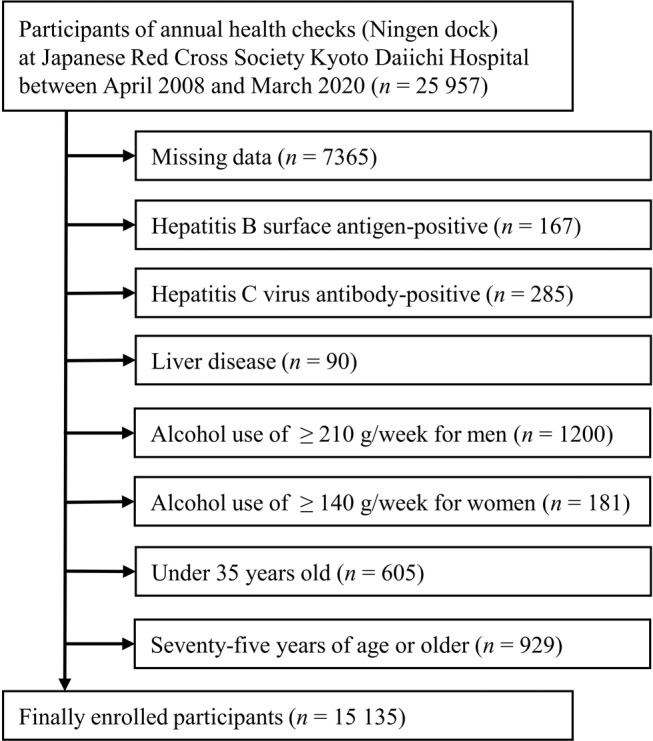
Flow diagram of the participants in the cross‐sectional study.

We performed a cross‐sectional study using a recruitment method with opt‐out option. The data of annual health checks (Ningen dock) were recorded at the Japanese Red Cross Society Kyoto Daiichi Hospital in Kyoto city, Japan. The Ningen dock is a voluntary, comprehensive health check system for early detection of chronic diseases in Japan.^21^ As shown in Figure 1, we used the first‐visit data of 25 957 participants (aged 20–93 years) between April 2008 and March 2020. Exclusion criteria were as follows: missing data (*n* = 7365); hepatitis B surface antigen‐ and hepatitis C virus antibody‐positive patients (*n* = 167 and 285, respectively); liver disease (*n* = 90); excessive alcohol use of ≥210 g/week for men and ≥140 g/week for women (*n* = 1200 and 181, respectively); and age <35 and ≥75 years (*n* = 605 and 929, respectively). Finally, we analyzed 15 135 participants (*n* = 7568 men and 7567 women) aged 35–74 years.

All procedures involving human participants were approved by the Ethical Committees of the Japanese Red Cross Society Kyoto Daiichi Hospital (approval number: 874) and Kyoto Prefectural University (approval number: 182). This study was conducted according to the guidelines laid down in the Declaration of Helsinki, and informed consent with opt‐out option was obtained from all participants.

### Measurements

Anthropometric and clinical measurements were performed as described previously.^22^ Body weight and height were obtained, and body mass index (BMI) was calculated as body weight (kg) divided by the square of height (m). Waist circumference was measured in the standing position at the umbilicus. Venous blood samples were collected after at least 12 h of fasting, and chemical analysis was performed using standard techniques. Aspartate aminotransferase (AST), alanine aminotransferase (ALT), and gamma‐glutamyl transpeptidase (γ‐GTP) were measured as markers of hepatic damage. Lipid and glucose metabolism were measured by total cholesterol, HDL cholesterol, low‐density lipoprotein (LDL) cholesterol, triglycerides, fasting plasma glucose, and hemoglobin A1c (HbA1c) National Glycohemoglobin Standardization Program (NGSP) values, which were calculated according to the standard equation of the Japan Diabetes Society (JDS): HbA1c (NGSP) % = 1.02 × HbA1c (JDS) % + 0.25%.^23^ C‐reactive protein (CRP) levels were used as markers of inflammation. The diagnosis of fatty liver was based on results of abdominal ultrasonography with hepatorenal contrast and liver brightness, performed by trained technicians. The evaluation of liver brightness, vascular blurring, hepatorenal echo contrast, and deep attenuation was performed, and a final diagnosis was made.^24^, ^25^ According to the criteria of MASLD,^19^, ^20^ non‐obese participants were divided into either the non‐MASLD or the MASLD group. The criteria were liver steatosis with at least one of five cardiometabolic risk factors: (i) BMI ≥ 25 kg/m^2^, waist circumference >94 cm in men and >80 cm in women; (ii) fasting glucose ≥100 mg/dL, HbA1c ≥5.7%, type 2 diabetes, or treatment for type 2 diabetes; (iii) systolic blood pressure ≥130 mmHg, diastolic blood pressure ≥85 mmHg, or specific antihypertensive drug use; (iv) triglycerides ≥150 mg/dL or lipid‐lowering treatment; and (v) HDL cholesterol ≤40 mg/dl in men and ≤ 50 mg/dL in women or lipid‐lowering treatment.

### Questionnaires

Lifestyle and dietary behaviors were assessed using a self‐administered questionnaire provided by the Health Department of the Ministry of Health, Labour and Welfare, Japan.^26^ The participants replied to the questionnaire regarding medical treatment, including heart disease, hypertension, hyperlipidemia, hyperuricemia, liver disease, and diabetes mellitus. Alcohol use was evaluated by asking the participants about the amount and type of alcoholic beverages consumed per week, which was used to estimate the mean ethanol intake per week. Habitual alcohol drinking was defined as the consumption of more than 140 g/week ethanol and drinking alcohol more than four times a week. Smoking habits were classified as never, former, and current smokers. Participants who exercised for more than 30 min at least twice a week for more than 1 year were regarded as having exercise habits. We included additional questions about dietary behaviors related to NAFLD and/or obesity.^27^ Participants were asked about their eating habits such as whether they consumed five food items (often eat vegetables, often eat soy products, often eat sweet buns/bread with fillings, often eat sesame/nuts, and often consume soft drinks), four food styles (often eat simmered/teriyaki food, often eat stir‐/deep‐fried food, often eat noodles/rice bowl, and often eat out/eat ready‐made food), and three dietary behaviors (often eat evening meal, fast eating, and consume ≥30 different food items per day). Applicability criteria for “often eat”, “often consume”, and “consume≥ 30 different food items per day” were three or more times a week.

### Statistical analysis

SPSS version 29.0 (SPSS Inc.) was used for statistical analyses. According to Asian criteria for BMI,^28^ the participants were divided into non‐obese (BMI < 25 kg/m^2^) and obese (BMI ≥ 25 kg/m^2^) groups. Sex was subdivided into men and women. Continuous variables were presented as means ± standard deviations, and categorical variables were expressed as numbers (%). Differences between the non‐NAFLD/MASLD and NAFLD/MASLD groups were assessed using the Mann–Whitney U test (continuous variables) and Chi‐squared test (categorical variables). Logistic regression analysis was performed to identify the risk factors for NAFLD and MASLD. Univariate and multivariable analyses were performed to examine the relationship between dietary behaviors and NAFLD/MASLD. Covariates of sex, age, BMI, smoking habits, exercise habits, drinking habits, and under medical treatment were adjusted. Multivariable stepwise logistic regression analysis was used to identify independent dietary factors associated with non‐obese NAFLD and MASLD. The existence of multicollinearity among covariates was investigated using Spearman's rank correlation coefficient. All dietary factors with statistical significance and covariates were included in the stepwise model. Significance was set at *P* < 0.05.

## Results

### NAFLD, MASLD, and obesity prevalence in this study

In the first‐visit data of 25 957 participants aged 20–93 years, 29.8% (*n* = 7739) had been diagnosed with NAFLD. NAFLD prevalence was higher in men (38.9%; *n* = 5005 out of 12 877) than in women (20.9%; *n* = 2734 out of 13 080). In the non‐obese participants, NAFLD was found in 22.0% overall (*n* = 4559 out of 20 709), with 30.0% men (*n* = 2863 out of 9543) and 15.2% women (*n* = 1696 out of 11 166). Non‐obese MASLD prevalence was 20.0% overall (*n* = 4140), with 26.8% men (*n* = 2557) and 14.2% women (*n* = 1583). Obese participants had higher prevalence of NAFLD in both men (64.3%; *n* = 2142 out of 3331) and women (56.0%; *n* = 1038 out of 1852).

For the 15 135 participants aged 35–74 years, the overall NAFLD prevalence was 34.1% (*n* = 5154), with higher prevalence in men (41.3%; *n* = 3124) than women (26.8%; *n* = 2030). As shown in Table 1, NAFLD was found in 1780 non‐obese men (31.0%) and 1344 obese men (74.0%). Similarly, NAFLD prevalence was lower in non‐obese women (19.4%; *n* = 1251) than obese women (70.6%: *n* = 779). In the non‐obese participants, MASLD was found in 1588 men (27.6%) and 1173 women (18.1%).

**Table 1:** Characteristics of non‐NAFLD and NAFLD Japanese participants according to obesity and sex differences.

	Non‐obese participants (BMI < 25 kg/m^2^)				Obese participants (BMI ≥ 25 kg/m^2^)			
	Men (*n* = 5751)		Women (*n* = 6464)		Men (*n* = 1817)		Women (*n* = 1103)	
	Non‐NAFLD	NAFLD	Non‐NAFLD	NAFLD	Non‐NAFLD	NAFLD	Non‐NAFLD	NAFLD
*n*	3971 (69.0%)	1780 (31.0%)	5213 (80.6%)	1251 (19.4%)	473 (26.0%)	1344 (74.0%)	324 (29.4%)	779 (70.6%)
Age (year)	56.4 ± 11.3	56.5 ± 10.4	55.5 ± 10.8	58.9 ± 9.3^†^	54.8 ± 11.4	54.1 ± 10.3	55.8 ± 10.9	58.0 ± 9.8^†^
Height (cm)	169.0 ± 6.2	169.2 ± 6.1	156.4 ± 5.7	155.5 ± 5.4^†^	169.4 ± 6.1	169.5 ± 6.1	154.8 ± 5.8	154.8 ± 5.7
Body weight (kg)	61.4 ± 7.0	65.6 ± 6.3^†^	50.0 ± 5.9	53.8 ± 5.3^†^	76.0 ± 7.6	79.5 ± 10.1^†^	64.1 ± 6.9	66.8 ± 8.6^†^
BMI (kg/m^2^)	21.5 ± 1.9	22.9 ± 1.5^†^	20.4 ± 2.1	22.3 ± 1.7^†^	26.5 ± 1.7	27.6 ± 2.7^†^	26.7 ± 1.9	27.8 ± 2.8^†^
WC (cm)	80.6 ± 6.2	85.1 ± 4.9^†^	76.8 ± 7.2	82.8 ± 6.4^†^	92.1 ± 6.0	95.3 ± 7.5^†^	90.9 ± 6.9	94.7 ± 7.8^†^
SBP (mmHg)	125.4 ± 16.6	127.6 ± 15.7^†^	119.9 ± 17.4	126.1 ± 17.3^†^	132.4 ± 16.4	133.2 ± 15.8	128.8 ± 18.0	134.0 ± 17.1^†^
DBP (mmHg)	77.0 ± 10.6	78.6 ± 10.2^†^	72.0 ± 11.1	75.4 ± 10.4^†^	81.6 ± 11.1	82.5 ± 10.5	76.6 ± 11.3	79.5 ± 11.3^†^
Blood metabolic parameters								
Triglyceride (mg/dL)	97.6 ± 60.4	133.4 ± 75.5^†^	79.6 ± 37.2	112.8 ± 60.7^†^	127.7 ± 88.6	154.3 ± 101.3^†^	96.7 ± 48.1	127.8 ± 64.3^†^
Total cholesterol (mg/dL)	201.9 ± 31.2	208.5 ± 32.9^†^	213.8 ± 34.2	220.5 ± 34.3^†^	203.4 ± 31.6	208.9 ± 35.4^†^	215.5 ± 33.4	219.5 ± 36.3
LDL cholesterol (mg/dL)	116.8 ± 27.4	125.5 ± 28.7^†^	120.5 ± 29.3	129.9 ± 30.4^†^	119.7 ± 28.3	127.6 ± 30.6^†^	128.3 ± 29.3	132.3 ± 32.9
HDL cholesterol (mg/dL)	59.5 ± 14.4	51.2 ± 11.7^†^	69.1 ± 14.7	60.3 ± 13.5^†^	53.5 ± 12.1	47.2 ± 10.0^†^	61.7 ± 12.7	56.5 ± 12.0^†^
Fasting glucose (mg/dL)	101.9 ± 17.6	106.9 ± 19.9^†^	95.0 ± 11.5	101.4 ± 16.7^†^	104.2 ± 16.8	112.2 ± 24.3^†^	98.9 ± 12.2	108.5 ± 24.0^†^
HbA1c (%)	5.8 ± 0.7	5.9 ± 0.8^†^	5.7 ± 0.5	5.9 ± 0.7^†^	5.8 ± 0.7	6.2 ± 1.0^†^	5.8 ± 0.7	6.2 ± 0.9^†^
AST (IU/L)	21.8 ± 8.6	22.9 ± 8.3^†^	20.6 ± 10.5	21.4 ± 8.9^†^	22.5 ± 7.6	27.0 ± 12.8^†^	19.6 ± 6.6	23.6 ± 10.2^†^
ALT (IU/L)	19.2 ± 11.2	25.9 ± 13.7^†^	15.9 ± 10.9	20.1 ± 14.8^†^	23.0 ± 13.2	37.1 ± 23.0^†^	17.1 ± 7.4	26.2 ± 16.5
γ‐GTP (IU/L)	38.5 ± 51.1	41.3 ± 35.0^†^	21.0 ± 19.1	26.5 ± 30.5^†^	48.8 ± 51.1	50.8 ± 41.6^†^	22.2 ± 16.5	32.7 ± 32.8^†^
CRP (mg/dL)	0.11 ± 0.37	0.13 ± 0.38^†^	0.08 ± 0.31	0.11 ± 0.23^†^	0.13 ± 0.38	0.18 ± 0.41^†^	0.13 ± 0.26	0.19 ± 0.39^†^
Smoking habits								
Never	1253 (31.6%)	530 (29.8%)^†^	4288 (82.3%)	1078 (86.2%)^†^	122 (25.8%)	411 (30.6%)	264 (81.5%)	646 (82.9%)
Past	1790 (45.1%)	913 (51.3%)	582 (11.2%)	106 (8.5%)	225 (47.6%)	623 (46.4%)	38 (11.7%)	81 (10.4%)
Current	928 (23.4%)	337 (18.9%)	343 (6.6%)	67 (5.4%)	126 (26.6%)	310 (23.1%)	22 (6.8%)	52 (6.7%)
Exercise habits	2437 (61.4%)	1036 (58.2%)^†^	2945 (56.5%)	752 (60.1%)^†^	254 (53.7%)	652 (48.5%)	164 (50.6%)	420 (53.9%)
Drinking habits	1841 (46.4%)	509 (28.6%)^†^	600 (11.5%)	42 (3.4%)^†^	247 (52.2%)	315 (23.4%)^†^	30 (9.3%)	24 (3.1%)^†^
Under medical treatment	1085 (27.3%)	602 (33.8%)^†^	1076 (20.6%)	424 (33.9%)^†^	184 (38.9%)	581 (43.2%)	111 (34.3%)	342 (43.9%)^†^

^†^ Significant difference between non‐NAFLD and NAFLD participants according to obesity and sex differences using Mann–Whitney U test (continuous variables) or Chi‐square test (categorical variables).

ALT, alanine aminotransferase; AST, aspartate aminotransferase; BMI, body mass index; CRP, C‐reactive protein; DBP, diastolic blood pressure; γ‐GTP, gamma‐glutamyl transpeptidase; HbA1c, glycated hemoglobin A1c; HDL, high‐density lipoprotein; LDL, low‐density lipoprotein; NAFLD, non‐alcoholic fatty liver disease; SBP, systolic blood pressure; WC, waist circumference.

The first‐visit data showed that obesity prevalence was 20.0% in the total participants, with 25.9% in men and 14.2% in women. In the analyzed data, obese participants were 19.3% in the total participants, with 24.0% in men and 14.6% in women.

### General and dietary characteristics of study participants

Anthropometric characteristics are shown in Table 1. Female participants with NAFLD were significantly older than those with no NAFLD. For both sex and BMI categories, NAFLD participants had significantly higher body weight, BMI, and WC compared to non‐NAFLD participants. Except for obese men, both systolic and diastolic blood pressure were significantly higher in NAFLD participants than non‐NAFLD participants. Blood metabolic parameters showed high levels of triglycerides, total cholesterol, LDL cholesterol, fasting glucose, HbA1c, AST, ALT, γ‐GTP, and CRP in NAFLD participants in both sex and BMI categories. HDL cholesterol was significantly lower in NAFLD participants than non‐NAFLD participants. There were significantly fewer current smokers in the non‐obese men and women with NAFLD than those with no NAFLD. The number of participants undergoing medical treatment was higher in the NAFLD participants than in non‐NAFLD participants.

Table 2 shows the dietary characteristics of the study participants. For non‐obese men, the NAFLD participants were more likely to often consume “soft drinks”, “noodles/rice bowl”, and “evening meal”, and have “fast eating” compared to non‐NAFLD participants. “Often eat sesame/nuts” was significantly lower in non‐obese NAFLD men than non‐obese non‐NAFLD men. Non‐obese NAFLD women were more likely to often eat “sweet buns/bread with fillings”, “simmered/teriyaki food”, “noodles/rice bowl”, “evening meal”, “fast eating”, and “consume ≥30 foods per day”. For obese men, significantly more NAFLD participants often eat “noodles/rice bowl”, “eating out/ready‐made food”, and “evening meal” than non‐NAFLD participants. There was no significant difference in dietary characteristics between obese non‐NAFLD and NAFLD women.

**Table 2:** Dietary characteristics of non‐NAFLD and NAFLD Japanese participants according to obesity and sex differences.

	Non‐obese participants (BMI < 25 kg/m^2^)				Obese participants (BMI ≥ 25 kg/m^2^)			
	Men (*n* = 5751)		Women (*n* = 6464)		Men (*n* = 1817)		Women (*n* = 1103)	
	Non‐NAFLD	NAFLD	Non‐NAFLD	NAFLD	Non‐NAFLD	NAFLD	Non‐NAFLD	NAFLD
*n*	3971 (69.0%)	1780 (31.0%)	5213 (80.6%)	1251 (19.4%)	473 (26.0%)	1344 (74.0%)	324 (29.4%)	779 (70.6%)
Food preferences								
Vegetables	2311 (58.2%)	987 (55.4%)	3662 (70.2%)	877 (70.1%)	258 (54.5%)	667 (49.6%)	217 (67.0%)	494 (63.4%)
Soybean products	1609 (40.5%)	707 (39.7%)	2418 (46.4%)	555 (44.4%)	172 (36.4%)	423 (31.5%)	140 (43.2%)	322 (41.3%)
Sweet buns/bread with fillings	800 (20.1%)	373 (21.0%)	1097 (21.0%)	309 (24.7%)^†^	104 (22.0%)	348 (25.9%)	83 (25.6%)	191 (24.5%)
Sesame/nuts	613 (15.4%)	228 (12.8%)^†^	1209 (23.2%)	293 (23.4%)	62 (13.1%)	189 (14.1%)	57 (17.6%)	155 (19.9%)
Soft drinks	607 (15.3%)	317 (17.8%)^†^	502 (9.6%)	127 (10.2%)	81 (17.1%)	285 (21.2%)	31 (9.6%)	105 (13.5%)
Food styles								
Simmered/teriyaki food	1142 (28.8%)	493 (27.7%)	2201 (42.2%)	586 (46.8%)^†^	139 (29.4%)	343 (25.5%)	127 (39.2%)	345 (44.3%)
Stir‐/deep‐fried food	1261 (31.8%)	589 (33.1%)	1192 (22.9%)	299 (23.9%)	200 (42.3%)	621 (46.2%)	104 (32.1%)	236 (30.3%)
Noodles/rice bowl	1287 (32.4%)	668 (37.5%)^†^	917 (17.6%)	254 (20.3%)^†^	165 (34.9%)	551 (41.0%)^†^	87 (26.9%)	187 (24.0%)
Eating out/ready‐made food	920 (23.2%)	444 (24.9%)	927 (17.8%)	247 (19.7%)	134 (28.3%)	466 (34.7%)^†^	89 (27.5%)	190 (24.4%)
Dietary behaviors								
Evening meal	1000 (25.2%)	560 (31.5%)^†^	1704 (32.7%)	453 (36.2%)^†^	132 (27.9%)	466 (34.7%)^†^	108 (33.3%)	286 (36.7%)
Fast eating	2112 (53.2%)	1072 (60.2%)^†^	2286 (43.9%)	661 (52.8%)^†^	325 (68.7%)	983 (73.1%)	202 (62.3%)	531 (68.2%)
Consume ≥30 foods per day	368 (9.3%)	148 (8.3%)	962 (18.5%)	263 (21.0%)^†^	36 (7.6%)	98 (7.3%)	64 (19.8%)	146 (18.7%)

^†^ Significant difference between non‐NAFLD and NAFLD participants according to obesity and sex differences: Chi‐square test.

BMI, body mass index; NAFLD, non‐alcoholic fatty liver disease.

The anthropometric and dietary characteristics of the non‐obese MASLD participants are summarized in Table S1. For both sex categories, non‐obese MASLD participants had significantly higher anthropometric variables and unfavorable metabolic parameters compared to the non‐MASLD participants. Unlike non‐obese NAFLD, there was no statistical significance in “often eat evening meal” between non‐obese women with non‐MASLD and MASLD. Other dietary characteristics showed similar statistical differences between non‐obese NAFLD and non‐obese MASLD groups.

### Associations of NAFLD and MASLD with dietary characteristics

The results of logistic regression analyses in non‐obese participants are shown in Table 3. In univariate analysis, non‐obese male NAFLD was significantly and negatively associated with “often eat sesame/nuts”, whereas often consume “soft drinks”, “noodles/rice bowl”, “evening meal”, and “fast eating” were significantly and positively associated with NAFLD in non‐obese men. After adjustment for age and BMI, a significant association between NAFLD and “fast eating” was not found in non‐obese men. Multivariable‐adjusted logistic regression analysis indicated that “often eat sesame/nuts” was negatively, and often eat “noodles/rice bowl” and “evening meal” were positively, associated with non‐obese NAFLD in men.

**Table 3:** Associations between NAFLD and dietary characteristics from multivariable‐adjusted logistic regression analyses in non‐obese Japanese men and women.

	Non‐obese participants (BMI < 25 kg/m^2^)					
	Univariate		Age‐ and BMI‐adjusted		Multivariable‐adjusted^†^	
	OR (95% CI)	*P*	OR (95% CI)	*P*	OR (95% CI)	*P*
Men (*n* = 5751)						
Food preferences						
Vegetables	0.89 (0.80–1.00)	0.052	0.92 (0.82–1.04)	0.204	0.94 (0.83–1.07)	0.330
Soybean products	0.97 (0.86–1.08)	0.568	0.96 (0.85–1.09)	0.513	0.98 (0.87–1.11)	0.774
Sweet buns/bread with fillings	1.05 (0.92–1.21)	0.482	1.07 (0.93–1.25)	0.341	0.98 (0.85–1.14)	0.830
Sesame/nuts	**0.81 (0.68–0.95)**	**0.009**	**0.77 (0.65–0.92)**	**0.004**	**0.78 (0.65–0.94)**	**0.007**
Soft drinks	**1.20 (1.04–1.39)**	**0.016**	**1.26 (1.07–1.47)**	**0.006**	1.09 (0.92–1.28)	0.319
Food styles						
Simmered/teriyaki food	0.95 (0.84–1.08)	0.409	0.93 (0.81–1.07)	0.321	0.96 (0.83–1.11)	0.567
Stir‐/deep‐fried food	1.06 (0.94–1.20)	0.317	0.91 (0.80–1.04)	0.170	0.94 (0.83–1.08)	0.385
Noodles/rice bowl	**1.25 (1.12–1.41)**	**<0.001**	**1.18 (1.04–1.33)**	**0.011**	**1.18 (1.04–1.35)**	**0.010**
Eating out/ready‐made food	1.10 (0.97–1.26)	0.143	1.06 (0.92–1.22)	0.415	1.07 (0.92–1.23)	0.391
Dietary behaviors						
Evening meal	**1.36 (1.21–1.54)**	**<0.001**	**1.30 (1.14–1.48)**	**<0.001**	**1.17 (1.02–1.34)**	**0.026**
Fast eating	**1.33 (1.19–1.49)**	**<0.001**	1.04 (0.92–1.17)	0.553	1.00 (0.88–1.13)	0.972
Consume ≥30 foods per day	0.89 (0.73–1.08)	0.243	0.87 (0.71–1.08)	0.215	0.87 (0.70–1.08)	0.203
Women (*n* = 6464)						
Food preferences						
Vegetables	0.99 (0.87–1.14)	0.921	0.96 (0.83–1.11)	0.607	0.94 (0.81–1.09)	0.403
Soybean products	0.92 (0.81–1.04)	0.198	0.88 (0.77–1.00)	0.053	**0.86 (0.75–0.99)**	**0.035**
Sweet buns/bread with fillings	**1.23 (1.07–1.42)**	**0.005**	**1.31 (1.12–1.53)**	**<0.001**	**1.26 (1.07–1.47)**	**0.004**
Sesame/nuts	1.01 (0.88–1.17)	0.863	0.92 (0.79–1.08)	0.298	0.91 (0.78–1.07)	0.235
Soft drinks	1.06 (0.86–1.30)	0.576	1.09 (0.87–1.36)	0.446	1.07 (0.85–1.34)	0.576
Food styles						
Simmered/teriyaki food	**1.21 (1.07–1.37)**	**0.003**	0.98 (0.85–1.12)	0.730	0.96 (0.83–1.10)	0.544
Stir‐/deep‐fried food	1.06 (0.92–1.23)	0.435	1.02 (0.87–1.19)	0.830	1.03 (0.88–1.21)	0.698
Noodles/rice bowl	**1.19 (1.02–1.39)**	**0.025**	1.11 (0.94–1.31)	0.237	1.15 (0.97–1.37)	0.099
Eating out/ready‐made food	1.14 (0.97–1.33)	0.106	1.14 (0.96–1.35)	0.132	1.18 (0.99–1.40)	0.062
Dietary behaviors						
Evening meal	**1.17 (1.03–1.33)**	**0.018**	**1.25 (1.09–1.44)**	**0.002**	**1.22 (1.06–1.40)**	**0.006**
Fast eating	**1.43 (1.27–1.62)**	**<0.001**	1.09 (0.95–1.24)	0.225	1.08 (0.94–1.24)	0.261
Consume ≥30 foods per day	**1.18 (1.01–1.37)**	**0.037**	1.08 (0.92–1.28)	0.350	1.09 (0.92–1.29)	0.338

^†^ Adjusted for age, BMI, smoking habits, exercise habits, drinking habits, and under medical treatment.

The results of a multivariable logistic regression are shown. Bold indicates significance at *P* < 0.05.

BMI, body mass index; CI, confidence interval; NAFLD, non‐alcoholic fatty liver disease; OR, odds ratio.

For non‐obese women, univariate odds ratios (ORs) of NAFLD were significantly and positively associated with often eat “sweet buns/bread with fillings”, “simmered/teriyaki food”, “noodles/rice bowl”, “evening meal”, “fast eating”, and “consume ≥30 foods per day”. The positive associations of often eat “sweet buns/bread with fillings” and “evening meal” remained significant after adjustment for age and BMI. Multivariable‐adjusted logistic regression analysis showed that often eat “sweet buns/bread with fillings” and “evening meal” were positively associated, whereas “often eat soybean products” was identified as a significant negative predictor of NAFLD in non‐obese women.

As shown in Table 4, univariate analysis found that obese NAFLD was significantly and positively associated with often eat “noodles/rice bowl”, “eating out/ready‐made food”, and “evening meal” in the men. For “often eat evening meal” in the obese men, the associations remained significant after adjustment for age and BMI. Unlike non‐obese participants, the significant association between each dietary characteristics and NAFLD was lost when adjusting for multivariable factors, including age, BMI, lifestyle habits, and under medical treatment. For obese women, no dietary characteristic was significantly associated with the risk of NAFLD in both univariate and adjusted models.

**Table 4:** Associations between NAFLD and dietary characteristics from multivariable adjusted logistic regression analyses in obese Japanese men and women.

			Obese participants (BMI ≥ 25 kg/m^2^)			
	Univariate		Age and BMI adjusted		Multivariable adjusted^†^	
	OR (95% CI)	*P*	OR (95% CI)	*P*	OR (95% CI)	*P*
Men (*n* = 1817)						
Food preferences						
Vegetables	0.82 (0.67–1.01)	0.066	0.88 (0.71–1.09)	0.250	0.84 (0.67–1.06)	0.133
Soybean products	0.80 (0.65–1.00)	0.051	0.81 (0.65–1.02)	0.069	0.79 (0.62–1.00)	0.053
Sweet buns/bread with fillings	1.24 (0.97–1.59)	0.091	1.24 (0.96–1.60)	0.102	1.08 (0.83–1.41)	0.581
Sesame/nuts	1.09 (0.80–1.48)	0.605	1.12 (0.82–1.53)	0.489	1.06 (0.77–1.47)	0.711
Soft drinks	1.30 (0.99–1.71)	0.058	1.24 (0.94–1.64)	0.131	1.05 (0.78–1.41)	0.757
Food styles						
Simmered/teriyaki food	0.82 (0.65–1.04)	0.102	0.90 (0.71–1.15)	0.417	0.88 (0.69–1.14)	0.344
Stir‐/deep‐fried food	1.17 (0.95–1.45)	0.141	1.06 (0.85–1.32)	0.617	1.07 (0.85–1.35)	0.561
Noodles/rice bowl	**1.30 (1.04–1.61)**	**0.019**	1.18 (0.95–1.48)	0.143	1.22 (0.97–1.55)	0.090
Eating out/ready‐made food	**1.34 (1.07–1.69)**	**0.012**	1.22 (0.96–1.54)	0.106	1.17 (0.91–1.49)	0.219
Dietary behaviors						
Evening meal	**1.37 (1.09–1.73)**	**0.007**	**1.32 (1.05–1.68)**	**0.020**	1.23 (0.96–1.58)	0.095
Fast eating	1.24 (0.99–1.56)	0.065	1.15 (0.91–1.46)	0.242	1.11 (0.87–1.42)	0.410
Consume ≥30 foods per day	0.96 (0.64–1.42)	0.819	1.03 (0.68–1.54)	0.901	1.14 (0.75–1.75)	0.539
Women (*n* = 1103)						
Food preferences						
Vegetables	0.86 (0.65–1.12)	0.261	0.83 (0.63–1.11)	0.208	0.85 (0.64–1.14)	0.287
Soybean products	0.93 (0.71–1.20)	0.565	0.87 (0.66–1.14)	0.317	0.87 (0.66–1.14)	0.303
Sweet buns/bread with fillings	0.94 (0.70–1.27)	0.701	0.92 (0.68–1.26)	0.607	0.89 (0.65–1.21)	0.447
Sesame/nuts	1.16 (0.83–1.63)	0.377	1.10 (0.78–1.56)	0.588	1.09 (0.77–1.54)	0.631
Soft drinks	1.47 (0.96–2.25)	0.073	1.52 (0.98–2.36)	0.059	1.53 (0.98–2.39)	0.059
Food styles						
Simmered/teriyaki food	1.23 (0.95–1.61)	0.120	1.13 (0.86–1.50)	0.388	1.14 (0.86–1.51)	0.365
Stir‐/deep‐fried food	0.92 (0.70–1.22)	0.555	0.90 (0.67–1.21)	0.478	0.89 (0.66–1.20)	0.441
Noodles/rice bowl	0.86 (0.64–1.16)	0.319	0.82 (0.60–1.11)	0.197	0.81 (0.59–1.10)	0.177
Eating out/ready‐made food	0.85 (0.64–1.14)	0.284	0.79 (0.58–1.08)	0.140	0.81 (0.59–1.10)	0.173
Dietary behaviors						
Evening meal	1.16 (0.88–1.53)	0.286	1.20 (0.90–1.59)	0.214	1.18 (0.89–1.58)	0.249
Fast eating	1.29 (0.99–1.70)	0.063	1.16 (0.88–1.54)	0.292	1.17 (0.89–1.55)	0.268
Consume ≥30 foods per day	0.94 (0.68–1.30)	0.697	0.92 (0.66–1.29)	0.625	0.95 (0.67–1.34)	0.767

^†^ Adjusted for age, BMI, smoking habits, exercise habits, drinking habits, and under medical treatment.

The results of a multivariable logistic regression are shown. Bold indicates significance at *P* < 0.05.

BMI, body mass index; CI, confidence interval; NAFLD, non‐alcoholic fatty liver disease; OR, odds ratio.

Table S2 shows the results of logistic regression analyses in non‐obese MASLD. For the non‐obese men, the statistical significances between dietary characteristics and MASLD were similar to those of non‐obese NAFLD. Multivariable‐adjusted analysis found that “often eat sesame/nuts” was negatively, and often eat “noodles/rice bowl” and “evening meal” were positively associated with non‐obese MASLD in the men. For non‐obese women, “often eat evening meal” was not significantly associated with MASLD in the univariate analysis, but was significantly and positively associated with MASLD in the adjusted model. The multivariable‐adjusted analysis indicated that often eat “sweet buns/bread with fillings” and “eating out/ready‐made food” were positively associated, whereas “often eat soybean products” was negatively associated with MASLD in non‐obese women.

### Multivariable‐adjusted stepwise logistic regression analysis between non‐obese NAFLD/MASLD and dietary characteristics

**Figure 2:**
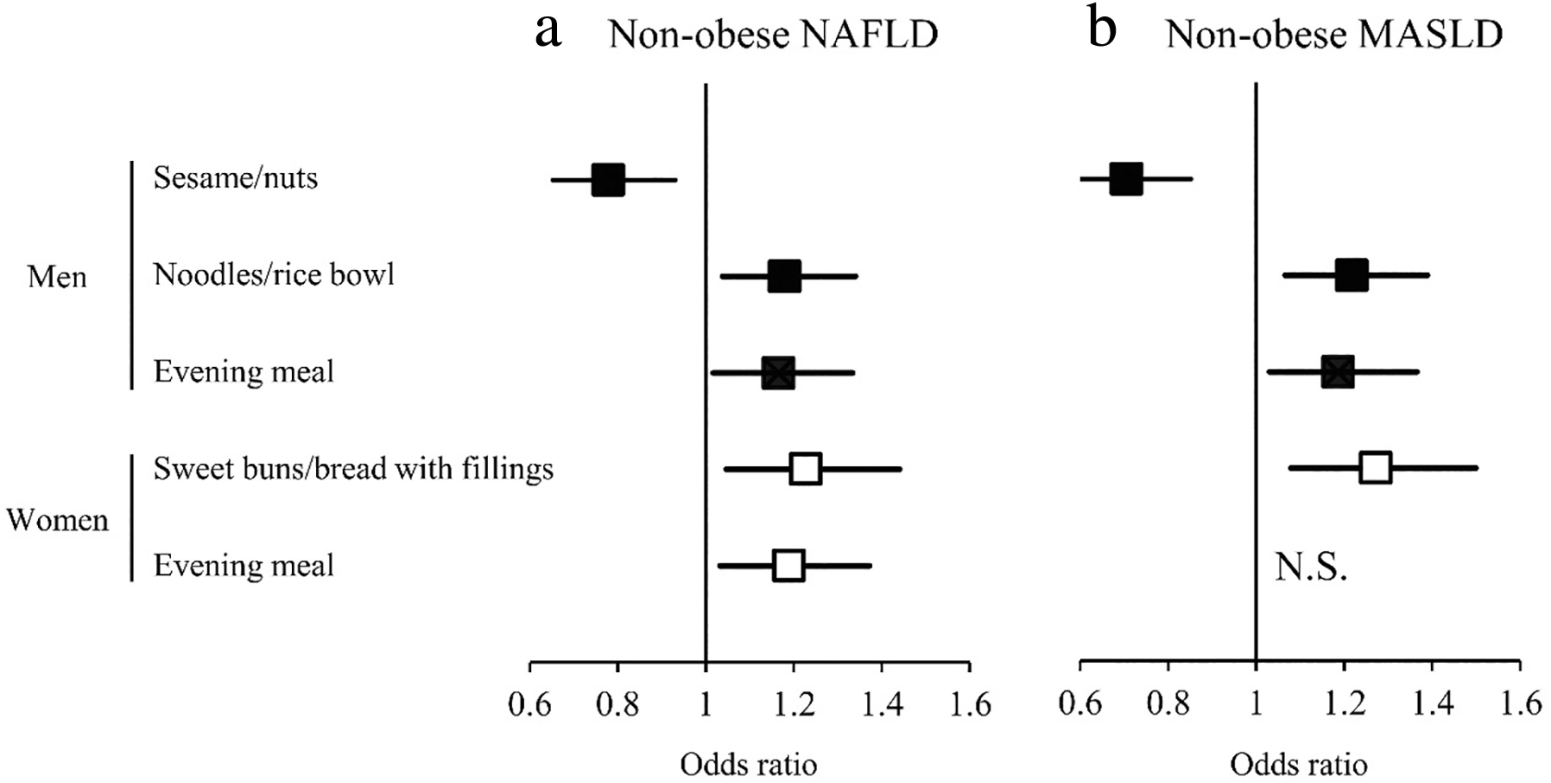
Associations of NAFLD and MASLD with dietary characteristics from multivariable‐adjusted stepwise logistic regression analysis in non‐obese Japanese men and women. (a) Associations between non‐obese NAFLD and dietary characteristics. (b) Associations between non‐obese MASLD and dietary characteristics. Squares indicate odds ratios, and horizontal lines indicate 95% confidence intervals. MASLD, metabolic dysfunction‐associated steatotic liver disease; NAFLD, non‐alcoholic fatty liver disease; N.S., not significant.

For non‐obese participants, the independent association between NAFLD and dietary characteristics was determined using multivariable‐adjusted stepwise logistic regression analysis (Fig. 2a). The analysis was performed according to sex, and the multivariable factors were the same as in non‐stepwise analyses. Stepwise analysis indicated that the ORs of non‐obese NAFLD males were significantly and negatively associated with “often eat sesame/nuts” (OR: 0.78, 95% confidence interval [CI]: 0.65–0.93). Positive predictors of NAFLD were often eat “noodles/rice bowl” (OR: 1.18, 95% CI: 1.04–1.34) and “evening meal” (OR: 1.17, 95% CI: 1.02–1.33) in the non‐obese men. For the non‐obese women, the ORs of NAFLD were significantly and positively associated with often eat “sweet buns/bread with fillings” (OR: 1.23, 95% CI: 1.05–1.44) and “evening meal” (OR: 1.19, 95% CI: 1.03–1.37).

Stepwise logistic regression analysis was performed to determine the association between non‐obese MASLD and dietary characteristics (Fig. 2b). The results showed that negative predictor of MASLD was “often eat sesame/nuts” (OR: 0.71, 95% CI: 0.59–0.85) in the non‐obese men, whereas the male ORs of non‐obese MASLD were significantly and positively associated with often eat “noodles/rice bowl” (OR: 1.22, 95% CI: 1.07–1.39) and “evening meal” (OR: 1.19, 95% CI: 1.03–1.37). The OR of MASLD was significantly and positively associated with “often eat sweet buns/bread with fillings” (OR: 1.27, 95% CI: 1.08–1.50) in the non‐obese women. For the non‐obese women, there was no significant association between “often eat evening meal” and MASLD in the stepwise analysis.

## Discussion

In this cross‐sectional study, we identified several NAFLD‐ and MASLD‐related dietary characteristics in the non‐obese participants. The dietary characteristics were partly similar but not identical between men and women. The results suggest that it is necessary to pay attention to sex differences in dietary characteristics to prevent non‐obese NAFLD and MASLD. On the other hand, multivariable‐adjusted analyses indicated that there was no significant association between obese NAFLD and dietary characteristics in both men and women. The present study did not perform quantitative analyses for dietary assessment; therefore, further studies are needed to examine whether dietary characteristics are related to the risk of NAFLD/MASLD including the obese population.

This study enrolled middle‐aged and older people with annual health checkup, and the obese population was about 5% less than national surveillance data.^29^ It is considered that the purpose of undergoing an annual health checkup is early detection of lifestyle‐related diseases such as cancer and metabolic disorders. There is a possibility that obese patients who had medical problems did not participate in this study, and the proportion of obese patients was less than the national data. Unexpectedly, the present study did not confirm the significant association between dietary characteristics and obese NAFLD. The overweight and obese population of Asian countries, including Japan, was reported to be lower than in Western countries.^30^ In addition, it was reported that the perception of being overweight is associated with trying to lose weight^31^, ^32^; thus, obesity itself may influence the association between NAFLD and dietary characteristics in middle‐aged and older Japanese. Moreover, in this study, over 40% of the obese NAFLD participants received medical treatment, which may have influenced dietary modification.

In a previous study, Asian individuals were found to have high prevalence of non‐obese NAFLD compared to Western individuals.^4^ In Japan, the prevalence of NAFLD increased from 25.0% in 2006–2010 to 29.6% in 2011–2016, and Japanese men have approximately twofold higher NAFLD prevalence than women.^7^ Our findings support the findings of these previous studies. For non‐obese NAFLD, a previous study performed in 2011–2012 reported that the prevalence of NAFLD was 15.2% in non‐obese Japanese,^33^ which was lower than the 22.0% reported in the present study. It may be considered that non‐obese NAFLD also has been increasing in Japan. As shown in a meta‐analysis^7^ and this study, NAFLD is associated with unfavorable metabolic parameters such as hypertension, hyperglycemia, and dyslipidemia, regardless of the BMI category. A previous study reported that high levels of CRP increased the risk of cardiovascular events and mortality^34^; thus, both unfavorable metabolic parameters and systemic inflammation might be associated with higher comorbidity and mortality in non‐obese NAFLD.^8^, ^9^ Previous studies considered that weight loss and lower BMI have a potential mortality risk in older people^35^; thus, it is important to evaluate independent factors of hepatic fat reduction, such as exercise^36^ and dietary characteristics, especially in the non‐obese NAFLD population.

This cross‐sectional study found some NAFLD‐related dietary characteristics in non‐obese participants. Sesame and nuts contain nutrients for lowering lipids, such as vitamin E^37^ and monounsaturated fatty acids.^38^ Previous studies have reported that the consumption of sesame reduced blood triglyceride levels^39^ and consumption of nuts and seeds was associated with a lower prevalence of NAFLD.^40^ It is therefore likely that the lipid‐lowering effects may be related to the lower NAFLD prevalence in non‐obese men who answered “often eat sesame/nuts”. Because blood triglycerides level, which is one of the main predictors of NAFLD,^41^ was lower in non‐obese women than men, the differences of lipid metabolism may explain why consumption of sesame and nuts was associated with lower NAFLD risk in the non‐obese men but not in the women. Habitual nighttime eating and snacking were associated with body weight gain and reduced whole body fat oxidation, respectively.^42^, ^43^ The present study found that “often eat evening meal” was an independent risk factor of non‐obese NAFLD in both men and women. Thus, the risk of NAFLD may be increased by the additional energy intake and lower lipid oxidation with evening meal. The risk of non‐obese NAFLD was found to increase with often eat “noodles/rice bowl” for men and “sweet buns/bread with fillings” for women, which were considered as a sex difference in food preference in Japan. The different main sources of excess energy intake may differ between non‐obese men and women.

Our secondary analysis, which evaluated the association between non‐obese MASLD and dietary characteristics, found the same significant association including often eat “sesame/nuts”, “noodles/rice bowl”, and “evening meal” in non‐obese men. The results suggest that these dietary characteristics were predictors of liver steatosis even in considering metabolic dysfunction. On the other hand, the results of stepwise regression analyses were slightly different between non‐obese women with NAFLD and MASLD. For non‐obese women, “often eat sweet buns/bread with fillings” was a positive predictor of both NAFLD and MASLD, whereas “often eat evening meal” was significantly associated with NAFLD but not with MASLD. There is a possibility that the evening meal was associated with hepatic fat accumulation, which was not accompanied with metabolic dysfunction, in some of the non‐obese women. Non‐obese MASLD was associated with three dietary characteristics in men and one dietary characteristic in women, and thus Japanese non‐obese men should pay more attention to dietary characteristics to prevent MASLD. As noted above, because obesity with NAFLD is classified as obese‐MASLD, this study found no significant association between obese‐MASLD and dietary characteristics.

Other dietary characteristics were not significantly related to NAFLD prevalence after multivariable adjustment in the present study. Low‐vegetable and high‐fat diets were a risk factor of NAFLD,^15^ but our study found that these dietary characteristics were similar between non‐NAFLD and NAFLD. It was reported that eating out was associated with increased total energy intake and energy contribution from fat.^44^ Dietary diversity and Japanese diet patterns, such as the consumption of simmered food, have beneficial effects on obesity and dyslipidemia.^45^, ^46^, ^47^ However, our logistic regression analyses did not indicate an association between these obesity‐related dietary characteristics and NAFLD prevalence. It was pointed out in a review that the risk of NAFLD increased by excess energy intake caused by the consumption of high‐fructose corn syrup, which is found in soft drinks.^48^ A previous study reported that half of non‐obese NAFLD adolescents regularly consumed soft drinks^17^; however, we found that approximately 10–20% of middle‐aged and older Japanese people were regular consumers. The changes in soft drink preference with age may be a reason why the independent association between NAFLD and soft drink consumption was not observed in the present study. Regarding eating speed, Cao *et al*. reported that although fast eating was significantly and positively associated with both obesity and NAFLD in Chinese adults, the significant association of NAFLD with fast eating was attenuated by adjustment for BMI and/or WC.^49^ Our findings support these findings; thus, fast eating may be more related to obesity than NAFLD in Asian populations.

This study has several limitations. First, we used a cross‐sectional design and a non‐quantitative questionnaire; thus, a causal relationship was not confirmed. A previous study reported that the risk of NAFLD was lowered by a nutrient‐rich diet,^50^ and thus further study is necessary to investigate the association between nutrient intake and non‐obese NAFLD/MASLD. Second, the diagnostic accuracy of ultrasonography for NAFLD was not considered.^51^ Third, the present study did not investigate other confounding factors such as NAFLD‐associated polymorphisms^52^ and gut microbiota.^53^ It was reported that synbiotics had beneficial effects on cardiovascular risk factors in NAFLD patients.^54^

## Conclusions

This cross‐sectional study found the existence of sex and obesity differences in the association of NAFLD and MASLD with dietary characteristics. In multivariable adjusted logistic regression analysis, some dietary characteristics were found to be associated with the NAFLD/MASLD prevalence in non‐obese participants, but not in obese participants. Our findings suggest the importance of healthy dietary choices to prevent non‐obese NAFLD and MASLD.

## Supporting information


**Table S1.** Characteristics of non‐MASLD and MASLD in non‐obese Japanese participants according to sex differences.
**Table S2.** Associations between MASLD and dietary characteristics from multivariable adjusted logistic regression analyses in non‐obese Japanese men and women.
