# Supplementary material for: Dietary characteristics associated with the risk of non‐alcoholic fatty liver disease and metabolic dysfunction‐associated steatotic liver disease in non‐obese Japanese participants: A cross‐sectional study
Source: JGH Open. 2024 May 22;8(5):e13082. doi: 10.1002/jgh3.13082 (PMC11109997; doi:10.1002/jgh3.13082)
Supplement: Supplementary file 1 — Table S1. Characteristics of non‐MASLD and MASLD in non‐obese Japanese participants according to sex differences. Table S2. Associations between MASLD and dietary characteristics from multivariable adjusted logistic regression analyses in non‐obese Japanese men and women. [file JGH3-8-e13082-s001.docx]

| Table S1. Characteristics of non-MASLD and MASLD in non-obese Japanese participants according to sex differences. | | | | | | | | | | | | | | | | |
| --- | --- | --- | --- | --- | --- | --- | --- | --- | --- | --- | --- | --- | --- | --- | --- | --- |
|  |  | Non-obese participants (BMI < 25 kg/m^2^) | | | | | | | | | | | | | | |
|  |  | Men (*n* = 5751) | | | | | | |  | Women (*n* = 6464) | | | | | | |
|  |  | Non-MASLD | | |  | MASLD | | |  | Non-MASLD | | |  | MASLD | | |
| *n* | | 4163 | (72.4%) | |  | 1588 | (27.6%) | |  | 5291 | (81.9%) | |  | 1173 | (18.1%) | |
| Age (year) | | 56.0 | ± | 11.3 |  | 57.5 | ± | 10.1* |  | 55.4 | ± | 10.8 |  | 59.6 | ± | 8.9* |
| Height (cm) | | 169.1 | ± | 6.2 |  | 168.9 | ± | 6.0 |  | 156.4 | ± | 5.7 |  | 155.4 | ± | 5.4* |
| Body weight (kg) | | 61.6 | ± | 7.1 |  | 65.6 | ± | 6.2* |  | 50.0 | ± | 5.9 |  | 54.1 | ± | 5.3* |
| BMI (kg/m^2^) | | 21.5 | ± | 1.9 |  | 22.9 | ± | 1.4* |  | 20.4 | ± | 2.1 |  | 22.4 | ± | 1.7* |
| WC (cm) | | 80.7 | ± | 6.1 |  | 85.4 | ± | 4.9* |  | 76.8 | ± | 7.2 |  | 83.4 | ± | 6.1* |
| SBP (mmHg) | | 125.0 | ± | 16.4 |  | 129.1 | ± | 15.7* |  | 119.7 | ± | 17.4 |  | 127.3 | ± | 17.0* |
| DBP (mmHg) | | 76.7 | ± | 10.5 |  | 79.5 | ± | 10.2* |  | 71.9 | ± | 11.0 |  | 76.0 | ± | 10.2* |
| Blood metabolic parameters | |  |  |  |  |  |  |  |  |  |  |  |  |  |  |  |
|  | Triglyceride (mg/dL) | 97.3 | ± | 59.3 |  | 138.8 | ± | 77.7* |  | 79.5 | ± | 37.1 |  | 115.5 | ± | 61.3* |
|  | Total cholesterol (mg/dL) | 202.0 | ± | 31.2 |  | 209.1 | ± | 33.1* |  | 213.6 | ± | 34.2 |  | 221.6 | ± | 34.3* |
|  | LDL cholesterol (mg/dL) | 117.1 | ± | 27.5 |  | 125.8 | ± | 28.8* |  | 120.4 | ± | 29.2 |  | 131.0 | ± | 30.3* |
|  | HDL cholesterol (mg/dL) | 59.4 | ± | 14.3 |  | 50.6 | ± | 11.6* |  | 69.1 | ± | 14.6 |  | 59.7 | ± | 13.5* |
|  | Fasting glucose (mg/dL) | 101.5 | ± | 17.3 |  | 108.5 | ± | 20.4* |  | 94.9 | ± | 11.4 |  | 102.2 | ± | 16.9* |
|  | HbA1c (%) | 5.7 | ± | 0.7 |  | 6.0 | ± | 0.8* |  | 5.7 | ± | 0.5 |  | 6.0 | ± | 0.8* |
|  | AST (IU/L) | 21.8 | ± | 8.5 |  | 23.1 | ± | 8.6* |  | 20.5 | ± | 10.5 |  | 21.5 | ± | 9.1* |
|  | ALT (IU/L) | 19.4 | ± | 11.2 |  | 26.2 | ± | 13.9* |  | 15.9 | ± | 10.8 |  | 20.5 | ± | 15.2* |
|  | γ-GTP (IU/L) | 38.2 | ± | 50.2 |  | 42.6 | ± | 35.8* |  | 20.9 | ± | 19.0 |  | 27.0 | ± | 31.2* |
|  | CRP (mg/dL) | 0.11 | ± | 0.37 |  | 0.13 | ± | 0.39* |  | 0.08 | ± | 0.31 |  | 0.11 | ± | 0.23* |
|  |  |  |  |  |  |  |  |  |  |  |  |  |  |  |  |  |
| Smoking habits | |  |  |  |  |  |  |  |  |  |  |  |  |  |  |  |
|  | Never | 1340 | (32.2%) | |  | 443 | (27.9%)* | |  | 4353 | (82.3%) | |  | 1013 | (86.4%)* | |
|  | Past | 1857 | (44.6%) | |  | 846 | (53.3%) | |  | 590 | (11.2%) | |  | 98 | (8.4%) | |
|  | Current | 966 | (23.2%) | |  | 299 | (18.8%) | |  | 348 | (6.6%) | |  | 62 | (5.3%) | |
| Exercise habits | | 2535 | (60.9%) | |  | 938 | (59.1%) | |  | 2978 | (56.3%) | |  | 719 | (61.3%)* | |
| Drinking habits | | 1889 | (45.4%) | |  | 461 | (29.0%)* | |  | 602 | (11.4%) | |  | 40 | (3.4%)* | |
| Under medical treatment | | 1089 | (26.2%) | |  | 598 | (37.7%)* | |  | 1076 | (20.3%) | |  | 424 | (36.1%)* | |
|  | |  |  | |  |  |  | |  |  |  | |  |  |  | |
| Food preferences | |  |  | |  |  |  | |  |  |  | |  |  |  | |
|  | Vegetables | 2409 | (57.9%) | |  | 889 | (56.0%) | |  | 3710 | (70.1%) | |  | 829 | (70.7%) | |
|  | Soybean products | 1675 | (40.2%) | |  | 641 | (40.4%) | |  | 2446 | (46.2%) | |  | 527 | (44.9%) | |
|  | Sweet buns/bread with fillings | 840 | (20.2%) | |  | 333 | (21.0%) | |  | 1118 | (21.1%) | |  | 288 | (24.6%)* | |
|  | Sesame/nuts | 644 | (15.5%) | |  | 197 | (12.4%)* | |  | 1222 | (23.1%) | |  | 280 | (23.9%) | |
|  | Soft drinks | 642 | (15.4%) | |  | 282 | (17.8%)* | |  | 510 | (9.6%) | |  | 119 | (10.1%) | |
| Food styles | |  |  | |  |  |  | |  |  |  | |  |  |  | |
|  | Simmered/teriyaki food | 1178 | (28.3%) | |  | 457 | (28.8%) | |  | 2230 | (42.1%) | |  | 557 | (47.5%)* | |
|  | Stir-/deep-fried food | 1326 | (31.9%) | |  | 524 | (33.0%) | |  | 1218 | (23.0%) | |  | 273 | (23.3%) | |
|  | Noodles/rice bowl | 1353 | (32.5%) | |  | 602 | (37.9%)* | |  | 934 | (17.7%) | |  | 237 | (20.2%)* | |
|  | Eating out/ready-made food | 976 | (23.4%) | |  | 388 | (24.4%) | |  | 943 | (17.8%) | |  | 231 | (19.7%) | |
| Dietary behaviors | |  |  | |  |  |  | |  |  |  | |  |  |  | |
|  | Evening meal | 1063 | (25.5%) | |  | 497 | (31.3%)* | |  | 1744 | (33.0%) | |  | 413 | (35.2%) | |
|  | Fast eating | 2222 | (53.4%) | |  | 962 | (60.6%)* | |  | 2324 | (43.9%) | |  | 623 | (53.1%)* | |
|  | Consume ≥ 30 foods per day | 382 | (9.2%) | |  | 134 | (8.4%) | |  | 974 | (18.4%) | |  | 251 | (21.4%)* | |
| ALT, alanine aminotransferase; AST, aspartate aminotransferase; BMI, body mass index; CRP, C-reactive protein; DBP, diastolic blood pressure; γ-GTP, gamma-glutamyl transpeptidase; HbA1c, glycated hemoglobin A1c; HDL, high density lipoprotein; LDL, low density lipoprotein; MASLD, metabolic dysfunction-associated steatotic liver disease; SBP, systolic blood pressure; WC, waist circumference. | | | | | | | | | | | | | | | | |
| * Significant difference between non-MASLD and MASLD patients according to sex difference using Mann–Whitney U test (continuous variables) or Chi-square test (categorical variables). | | | | | | | | | | | | | | | | |

| Table S2. Associations between MASLD and dietary characteristics from multivariable adjusted logistic regression analyses in non-obese Japanese men and women. | | | | | | | | | | | | | |
| --- | --- | --- | --- | --- | --- | --- | --- | --- | --- | --- | --- | --- | --- |
|  |  |  | Non-obese participants (BMI < 25 kg/m^2^) | | | | | | | | | | |
|  |  |  | Univariate | | |  | Age and BMI adjusted | | |  | Multivariable adjusted* | | |
|  |  |  | OR | (95% CI) | *P* |  | OR | (95% CI) | *P* |  | OR | (95% CI) | *P* |
| Men (*n* = 5751) | | |  |  |  |  |  |  |  |  |  |  |  |
|  | Food preferences | |  |  |  |  |  |  |  |  |  |  |  |
|  |  | Vegetables | 0.93 | (0.82-1.04) | 0.196 |  | 0.92 | (0.81-1.04) | 0.188 |  | 0.94 | (0.83-1.07) | 0.372 |
|  |  | Soybean products | 1.01 | (0.89-1.13) | 0.928 |  | 0.96 | (0.84-1.09) | 0.510 |  | 0.99 | (0.87-1.13) | 0.890 |
|  |  | Sweet buns/bread with fillings | 1.05 | (0.91-1.21) | 0.505 |  | 1.09 | (0.94-1.27) | 0.266 |  | 1.00 | (0.86-1.17) | 0.973 |
|  |  | Sesame/nuts | **0.77** | **(0.65-0.92)** | **0.003** |  | **0.71** | **(0.59-0.85)** | **<0.001** |  | **0.72** | **(0.59-0.86)** | **<0.001** |
|  |  | Soft drinks | **1.18** | **(1.02-1.38)** | **0.031** |  | **1.29** | **(1.09-1.52)** | **0.003** |  | 1.12 | (0.94-1.33) | 0.202 |
|  | Food styles | |  |  |  |  |  |  |  |  |  |  |  |
|  |  | Simmered/teriyaki food | 1.02 | (0.90-1.16) | 0.717 |  | 0.93 | (0.81-1.08) | 0.338 |  | 0.96 | (0.83-1.11) | 0.578 |
|  |  | Stir-/deep-fried food | 1.05 | (0.93-1.19) | 0.406 |  | 0.95 | (0.83-1.08) | 0.431 |  | 0.97 | (0.85-1.11) | 0.678 |
|  |  | Noodles/rice bowl | **1.27** | **(1.12-1.43)** | **<0.001** |  | **1.22** | **(1.07-1.38)** | **0.003** |  | **1.20** | **(1.06-1.38)** | **0.006** |
|  |  | Eating out/ready-made food | 1.06 | (0.92-1.21) | 0.431 |  | 1.07 | (0.92-1.23) | 0.393 |  | 1.06 | (0.91-1.23) | 0.435 |
|  | Dietary behaviors | |  |  |  |  |  |  |  |  |  |  |  |
|  |  | Evening meal | **1.33** | **(1.17-1.51)** | **<0.001** |  | **1.30** | **(1.14-1.49)** | **<0.001** |  | **1.18** | **(1.02-1.35)** | **0.024** |
|  |  | Fast eating | **1.34** | **(1.19-1.51)** | **<0.001** |  | 1.06 | (0.93-1.20) | 0.388 |  | 1.01 | (0.89-1.15) | 0.858 |
|  |  | Consume ≥ 30 foods per day | 0.91 | (0.74-1.12) | 0.382 |  | 0.85 | (0.68-1.05) | 0.135 |  | 0.85 | (0.67-1.06) | 0.146 |
|  |  |  |  |  |  |  |  |  |  |  |  |  |  |
| Women (*n* = 6464) | | |  |  |  |  |  |  |  |  |  |  |  |
|  | Food preferences | |  |  |  |  |  |  |  |  |  |  |  |
|  |  | Vegetables | 1.03 | (0.89-1.18) | 0.707 |  | 0.97 | (0.83-1.13) | 0.715 |  | 0.95 | (0.81-1.11) | 0.493 |
|  |  | Soybean products | 0.95 | (0.84-1.08) | 0.418 |  | 0.88 | (0.76-1.01) | 0.064 |  | **0.86** | **(0.75-1.00)** | **0.043** |
|  |  | Sweet buns/bread with fillings | **1.22** | **(1.05-1.41)** | **<0.001** |  | **1.32** | **(1.12-1.55)** | **0.001** |  | **1.27** | **(1.08-1.50)** | **0.004** |
|  |  | Sesame/nuts | 1.04 | (0.90-1.21) | 0.570 |  | 0.92 | (0.78-1.09) | 0.331 |  | 0.91 | (0.77-1.07) | 0.252 |
|  |  | Soft drinks | 1.06 | (0.86-1.31) | 0.597 |  | 1.12 | (0.89-1.42) | 0.323 |  | 1.10 | (0.87-1.39) | 0.428 |
|  | Food styles | |  |  |  |  |  |  |  |  |  |  |  |
|  |  | Simmered/teriyaki food | **1.24** | **(1.09-1.41)** | **<0.001** |  | 0.95 | (0.82-1.09) | 0.441 |  | 0.93 | (0.80-1.07) | 0.309 |
|  |  | Stir-/deep-fried food | 1.01 | (0.87-1.18) | 0.852 |  | 0.99 | (0.84-1.17) | 0.886 |  | 1.00 | (0.85-1.18) | 0.991 |
|  |  | Noodles/rice bowl | **1.18** | **(1.01-1.39)** | **0.040** |  | 1.09 | (0.92-1.30) | 0.321 |  | 1.14 | (0.95-1.36) | 0.155 |
|  |  | Eating out/ready-made food | 1.13 | (0.96-1.33) | 0.133 |  | 1.16 | (0.97-1.38) | 0.097 |  | **1.20** | **(1.00-1.43)** | **0.047** |
|  | Dietary behaviors | |  |  |  |  |  |  |  |  |  |  |  |
|  |  | Evening meal | 1.11 | (0.97-1.26) | 0.140 |  | **1.21** | **(1.04-1.40)** | **0.012** |  | **1.18** | **(1.02-1.36)** | **0.030** |
|  |  | Fast eating | **1.45** | **(1.27-1.64)** | **<0.001** |  | 1.08 | (0.94-1.24) | 0.272 |  | 1.07 | (0.93-1.23) | 0.331 |
|  |  | Consume ≥ 30 foods per day | **1.21** | **(1.03-1.41)** | **0.018** |  | 1.08 | (0.91-1.28) | 0.411 |  | 1.08 | (0.91-1.28) | 0.400 |
| BMI, body mass index; CI, confidence interval; MASLD, metabolic dysfunction-associated steatotic liver disease; OR, odds ratio.  The results of a multivariable logistic regression are shown. Bold indicates significance at P < 0.05.  *Adjusted for age, BMI, smoking habits, exercise habits, drinking habits, and under medical treatment. | | | | | | | | | | | | | |
